# Aggregation by peptide conjugation rescues poor immunogenicity of the HA stem

**DOI:** 10.1371/journal.pone.0241649

**Published:** 2020-11-02

**Authors:** Wenbo Jiang, Emily H. Pilkington, Hannah G. Kelly, Hyon-Xhi Tan, Jennifer A. Juno, Adam K. Wheatley, Stephen J. Kent

**Affiliations:** 1 Department of Microbiology and Immunology, Peter Doherty Institute for Infection and Immunity, University of Melbourne, Melbourne, Victoria, Australia; 2 Melbourne Sexual Health Clinic and Infectious Diseases Department, Alfred Hospital, Monash University Central Clinical School, Carlton, Victoria, Australia; 3 ARC Centre for Excellence in Convergent Bio-Nano Science and Technology, University of Melbourne, Melbourne, Australia; University of South Dakota, UNITED STATES

## Abstract

Influenza virus infection is a global public health threat. Current seasonal influenza vaccines are efficacious only when vaccine strains are matched with circulating strains. There is a critical need for developing “universal” vaccines that protect against all influenza viruses. HA stem is a promising target for developing broad-spectrum influenza vaccines due to its relatively conserved feature. However, HA stem is weakly immunogenic when administered alone in a soluble form. Several approaches have been employed to improve the immunogenicity of HA stem, including conjugation of HA stem with a highly immunogenic carrier protein or displaying HA stem on a nanoparticle scaffold. Converting a weakly immunologic protein into a multimer through aggregation can significantly enhance its immunogenicity, with some multimeric protein aggregates previously shown to be more immunogenic than their soluble counterparts in animal models. Here, we show that a chemically coupling a peptide derived from the head domain of PR8 HA (P35) with the poorly immunogenic HA stem protein results in aggregation of the HA stem which significantly increases stem-specific B cell responses following vaccination. Importantly, vaccination with this conjugate in the absence of adjuvant still induced robust B cell responses against stem in vivo. Improving HA stem immunogenicity by aggregation provides an alternative avenue to conjugation with exotic carrier proteins or nanoparticle formulation.

## Introduction

Seasonal influenza viruses circulate in all geographical regions of the world and cause recurrent disease in humans. Worldwide, these annual epidemics are estimated to cause 1 billion infections, with 3–5 million cases of severe illness and 290,000–650,000 deaths [1]. Influenza viruses undergo continual antigenic evolution allowing mutant viruses to evade host immunity acquired to previous virus strains. Current seasonal influenza vaccines are effective when vaccine strains are matched with circulating strains [2, 3]. However, there is little to no cross-protection against antigenic variants, emerging pandemic or zoonotic outbreak strains [4]. There is therefore tremendous interest in the development of novel “universal” vaccines, that would significantly expand protection to most or all influenza viruses.

HA is the most important surface glycoprotein which mediates viral entry into host cells [5, 6]. Influenza HA can be divided into two domains based on their functions [7]. The head domain contains the receptor-binding site via which the virus binds sialic acid receptors of the target cell, while the stem domain contains fusion peptide which mediates fusion between the viral envelope and the endosomal membrane of the host cell [5]. Unlike the head domain which is highly variable, the stem domain is relatively conserved among influenza viruses [7]. Additionally, anti-stem antibodies have been shown to provide cross-protection against both heterologous and heterosubtypic strains in mice [8, 9]. All these factors make the stem domain an attractive target for universal influenza vaccine. However, soluble stem protein is a poor immunogen which elicits weak humoral and cellular immune responses in vivo [10]. Antibodies are mainly generated against the immunodominant HA head domain in the context of full-length HA [10, 11]. Tan et al. in our group demonstrated that poor intrinsic immunogenicity was responsible for the weak antibody responses targeting HA stem [10].

Immunogen multimerization is an effective strategy for enhancing the immunogenicity of protein antigens [12]. Multimeric antigens can be classified into two major types: highly ordered nanoparticles and amorphous protein aggregates. The ability of nanoparticles to enhance the immunogenicity of protein antigens has been well demonstrated [13]. To overcome the poor immunogenicity of HA stem, Yassine et al. fused the gene encoding ferritin from Helicobacter pylori bacteria to H1 HA stem and expressed nanoparticles of eight trimeric HA stem molecules in their native confirmation around a spherical ferritin core [14]. This ferritin-stem nanoparticle vaccine induced broadly cross-reactive antibody responses and conferred full protection against heterosubtypic H5 virus challenge in mice after sequential immunizations [14].

Protein aggregates are high molecular weight proteins composed of multimers of native or denatured monomers [15]. Although the effect of protein aggregates on the immunogenicity of protein antigens has been assessed in a limited number of studies, experiments conducted in mice have demonstrated that multimeric protein aggregates are more immunogenic than the monomeric forms [16–18]. Hermeling et al. generated recombinant human interferon alpha2a/b (rhIFNα2) aggregates by metal-catalyzed oxidation and showed that the aggregated form increased rhIFNα2-specific antibody responses in wild-type FVB/N mice compared with the native form [17]. They also found that rhIFNα2-specific serum IgG titre is dependent on the level of aggregation [17]. Wei et al. showed that oligomeric aggregates of influenza HA expressed in mammalian cells induced significantly higher levels of neutralizing antibodies in BALB/c mice than monomeric or trimeric HA expressed in the same cell line [19]. Ilyinskii et al. fused a weakly immunogenic model antigen GFP with long polyglutamine (polyQ) domain that triggers protein aggregation and immunized BALB/c mice with plasmids encoding polyQ-GFP or GFP alone [18]. They found that plasmids encoding polyQ-GFP elicited significant higher anti-GFP antibody titre and anti-GFP CTL activity than plasmids encoding GFP alone [18]. Of note, the most widely used aluminium adjuvants also form fibrous loose aggregates of aluminium nanocrystals [20, 21]. Adsorption of antigens to aluminium aggregates enhances vaccine induced immune response [22]. In the present study, we show that conjugation of HA stem with a short HA head peptide rescues the poor immunogenicity of stem protein. We chose a 17-mer peptide from the HA head (termed P35) since we surmised that P35 might constitute a T-helper epitope, although this subsequently was disproven (shown below). Instead of being a T-helper epitope, P35 conjugation induces the formation of stem aggregates, which significantly enhances stem-specific B cell responses in mice.

## Materials and methods

### Mouse infection and immunization

Mouse studies and related experimental procedures in this study were approved by the University of Melbourne Animal Ethics Committee (#1914874). Female C57BL/6 mice (6–8 weeks old) were anesthetized by isoflurane inhalation prior to infection or immunization. For intranasal influenza infections, mice were instilled with 50μL of 50 TCID50 of A/Puerto Rico/8/34 (PR8) as described previously [10]. For intramuscular vaccinations, 5μg of PR8 HA stem protein [10], HIV gp120 protein (NIH AIDS Reagent Program), hen egg lysozyme (HEL) protein (Sigma), chicken egg ovalbumin (OVA) protein (InvivoGen) or their respective conjugates with peptides (refer to peptide and protein conjugation) with or without Addavax (1:1 ratio; InvivoGen) were injected into both hind quadriceps using a 29G needle. Fourteen days post infection or vaccination, draining lymph nodes (mediastinal lymph nodes (mLN) for infected animals, inguinal lymph nodes (inLN) and iliac lymph nodes (ilLN) for vaccinated animals) and blood were collected for FACS and ELISA analyses.

### Antigen stimulations and cell culture

Draining lymph nodes from 5 PR8-infected mice were mashed into single cell suspensions, pooled and cultured in RPMI 1640 supplemented with 10% fetal calf serum and penicillin/streptomycin (RF10). To identify antigen-specific Tfh cells using peptide stimulation [23], cell suspensions were stimulated for 18 hours in RF10 with PR8 HA peptide 11 (P11, LKGIAPLQLGKCNIAGW, 2μg/mL, BEI resources), PR8 HA peptide 35 which is located at positions 192–208 (H3 numbering) (P35, QNLYQNENAYVSVVTSN, 2μg/mL, BEI resources), Concanavalin A (Con A, 5ug/mL, Sigma) or a vehicle (DMSO) control. Cells were cultured in a 48-well plate in 500μl RF10 at a concentration of 2 million cells/ml. At the time of stimulation, anti-CD154 PE antibody (MR1; BioLegend) was added to all culture conditions.

### Peptide and protein conjugation

Peptide 11-cysteine (P11C, LKGIAPLQLGKCNIAGWC, GenScript) or peptide 35-cysteine (P35C, QNLYQNENAYVSVVTSNC, GenScript or LifeTein) were conjugated with PR8 HA stem protein [10], HIV gp120 protein (NIH AIDS Reagent Program), HEL protein (Sigma) or OVA protein (InvivoGen) using a commercial cross-linker, sulfosuccinimidyl 4-[N-maleimidomethyl]cyclohexane-1-carboxylate (Sulfo-SMCC, Thermo), according to the manufacturer’s instructions. Briefly, proteins were diluted with PBS to 1mg/ml and Sulfo-SMCC were dissolved in distilled water to 5mg/ml immediately before use. 20-fold molar excess of Sulfo-SMCC solution were then added to protein solution and reaction mixture were incubated for 30 minutes at room temperature. Excess Sulfo-SMCC was then removed using desalting columns (Thermo). Peptides were dissolved in DMSO to the desired concentration immediately before use and equal amounts of peptides (peptide: protein = 1:1, m/m) were then added to maleimide-activated proteins and the reaction mixtures were incubated for 30 minutes at room temperature. Excess peptides were then removed using Amicon Ultra Centrifugal Filter (Millipore, 10KD). For some experiments, P35C-stem conjugates were further filtered through Costar Spin-X Centrifuge Tube Filters (Corning, 0.22 μm) and purified by gel filtration using Superose 6 column (GE Life Sciences). The concentration of all peptide-protein conjugates was measured using Qubit 2.0 Fluorometer (Thermo).

### Flow cytometry

For detection of antigen-specific GC B cells, freshly isolated LN cell suspensions from individual mice were stained with Aqua Viability Dye (Thermo) and Fc blocked with an anti-CD16/32 antibody (clone 93; BioLegend). Cells were then surface stained with the relevant probes (APC or PE labelled stem, HEL, OVA or gp120 proteins for baiting antigen-specific GC B cells) [10] and the following antibodies: CD45 APC-Cy7 (30-F11; BD), CD3 BV785 (145-2C11; BioLegend), F4/80 BV785 (BM8; BioLegend), Streptavidin BV785 (BD), B220 BUV737 (RA3-6B2; BD), IgD BUV395 (11-26c.2a; BD), CD38 PE-Cy7 (90; BioLegend), GL7 AF488 (GL7; BioLegend). For detection of Tfh cells *ex vivo*, freshly isolated LN cell suspensions from individual mice were stained with the following panel: Live/dead Red (Thermo), B220 BV605 (RA3-6B2; BD Biosciences), CD3 BV510 (145-2C11; BioLegend), CD4 BUV737 (RM4-5; BD Biosciences), CXCR5 BV421 (L138D7; BioLegend), PD-1 BV786 (29F.1A12; BioLegend). For Ag-specific Tfh identification [23], cells were cultured as described above and then stained with the following panel: Live/dead Red (Thermo), B220 BV605 (RA3-6B2; BD), CD3 BV510 (145-2C11; BioLegend), CD4 BUV737 (RM4-5; BD), CD25 BB515 (PC61; BD), OX40 PeCy7 (OX-86; BioLegend). All samples were acquired on a BD LSR Fortessa using BD FACS Diva and data was analyzed in FlowJo v10.

### ELISA

Stem-specific IgG titre of serum from PR8 HA stem or peptide-stem conjugates immunized mice were detected using sandwich ELISA as previously described [24]. Briefly, 0.1μg/ml rabbit anti-His pAb (Genscript) was coated onto MaxiSorp plates (Thermo) overnight at 4°C. Plates were blocked with 5% skim milk powder in PBS before adding His-tagged PR8 HA stem protein [10]. Serially diluted mouse sera were then added to plates and incubated for 2 h at room temperature. Plates were washed prior to incubation with 1:5,000 dilution of HRP-conjugated goat anti-mouse IgG (Sera-Care) for 1 h at room temperature. Plates were washed and developed using TMB substrate (Sigma), reaction was stopped with 0.16M H_2_SO_4_ and read at 450nm. Endpoint titers were calculated as the reciprocal serum dilution giving signal 2x background using a fitted curve (4-parameter log regression).

The antigenicity of PR8 HA stem and P35C-stem conjugate was assessed with a panel of anti-HA stem monoclonal antibodies (CR9114, TN1F11, TN1G09, produced in-house) and a negative control antibody VRC01 (produced in-house) as preciously described [24]. Briefly, 96-well MaxiSorp plates (Thermo) were coated with PR8 HA stem and P35C-stem conjugate (2 μg/ml × 100 μl/well) overnight at 4°C. After blocking with 2.5% BSA in PBS, a panel of anti-HA stem monoclonal antibodies and a negative control antibody at different dilutions (starting at 1 μg/ml, four-fold serial dilutions) were added and incubated for two hours at room temperature. Plates were washed with 0.05% Tween 20 in PBS prior to incubation with 1:10,000 dilution of HRP-conjugated rabbit anti-human IgG (Dako) for 1 h at room temperature. Plates were washed again, developed using 3,3′,5,5′-Tetramethylbenzidine (TMB) substrate (Sigma), stopped with 0.16M H_2_SO_4_ and read at 450 nm.

### Dynamic light scattering and zeta potential

Dynamic light scattering and zeta potential measurements were performed on a Zetasizer Nano ZS (Malvern) instrument and analysed with Zetasizer software. Samples kept on ice were allowed to equilibrate to room temperature prior to analysis, with all measurements taking place at 25°C. Concentration of all samples was 0.38 mg/mL in PBS for light scattering experiments, and in Milli-Q water (pH 7) for zeta potential experiments to limit salt interference with measurements. Data represent mean values plus/minus standard deviation of three measurements each consisting of 12 runs.

### Statistical analysis

Data is presented as mean ± SEM. Statistical significance was assessed by one-way ANOVA and performed using GraphPad Prism version 7 (GraphPad Software). P values less than 0.05 were considered statistically significant and marked with one asterisk, P values less than 0.01 were marked with two asterisks, P values less than 0.001 were marked with three asterisks.

## Results

### P35-cysteine conjugation improves the immunogenicity of stem

The HA stem is relatively conserved and an attractive target for improved influenza vaccines. However, the HA stem is relatively poorly immunogenic. To study whether the immunogenicity of the HA stem could be improved, we conjugated peptides (P35 and P11) from HA head domain with HA stem proteins and evaluated the immune responses induced by various peptide-stem conjugates in mice. The P35 or P11 peptides and HA stem proteins were coupled with a crosslinker, Sulfo-SMCC. Sulfo-SMCC is a heterobifunctional crosslinker that contains N-hydroxysuccinimide (NHS) ester and maleimide group. The NHS ester reacts with primary amine from N-termimal or lysine of the protein and maleimide group reacts with sulfhydryl from cysteine of the peptide. Therefore, we added a cysteine at the C-terminal of the peptide during peptide synthesis (to produce P11C or P35C peptides). Antigenicity of the stem on peptide-stem conjugate is not altered, which is demonstrated by the similar binding activity of HA stem and P35C-stem with anti-HA stem monoclonal antibodies (CR9114, TN1F11 and TN1G09, S1 Fig). Next, we intramuscularly immunized mice with 5ug stem, or P11C-stem conjugate or P35C-stem conjugate or mixture of 5ug stem plus 5ug free P35C on day 0. Draining lymph nodes (dLNs) and blood were collected on day 14 post immunization.

**Fig 1:**
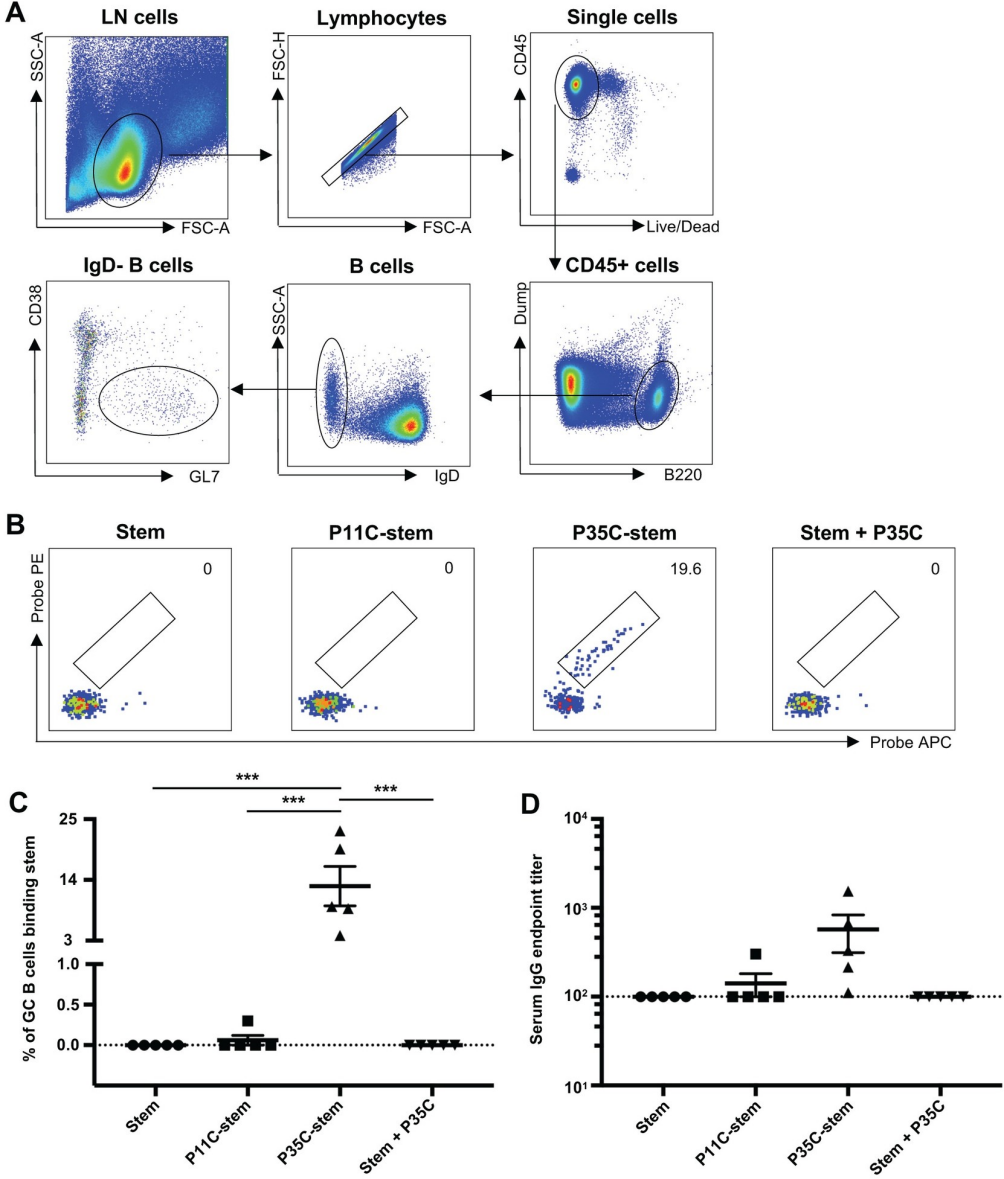
Stem-specific B cell and serological responses in immunized mice. (A) Gating strategy for GC B cells. Lymphocytes were identified by forward scatter area (FSC-A) and side-scatter area (SSC-A). Doublets were excluded by gating on single cells as determined by FSC-A versus FSC-H, and live cells were identified by viability dye exclusion. B cells were identified by B220+ and naïve B cells were excluded by IgD+. GC B cells were identified by GL7+CD38lo. For each step, the parental population is indicated above the plot. (B—C) Representative flow cytometric plots (B) and frequency (C) of GC B cells (B220+IgD- CD38lo GL7+) from mice (n = 5) immunized with adjuvanted (Addavax) immunogens double stained with PR8 HA stem probes. (D) Serum endpoint total IgG titres of mice (n = 5) immunized with adjuvanted (Addavax) immunogens. Data indicate the mean ± SEM. Statistical significance was assessed by one-way ANOVA.

We first analysed GC B cells for the proportion that were HA stem-specific using fluorescent stem probes [10]. The gating strategy for GC B cells is shown in Fig 1A. As expected, we found that stem was a poor immunogen which elicited negligible stem-specific GC B cell responses (Fig 1B and 1C). The P11C-stem conjugate, or a mixture of stem and free P35C, displayed similarly poor immunogenicity as the HA stem alone. In contrast, a striking proportion of GC B cells were specific for HA stem in the P35C-stem immunized mice (mean 12.9%, range 3.94%-22.9%). We next studied whether the enhanced generation of stem-specific GC B cells translated into the generation of enhanced stem specific antibodies. We found that stem-specific serum IgG was higher in the P35C-stem mice than that of other 3 stem immunized groups (Fig 1D).

**Fig 2:**
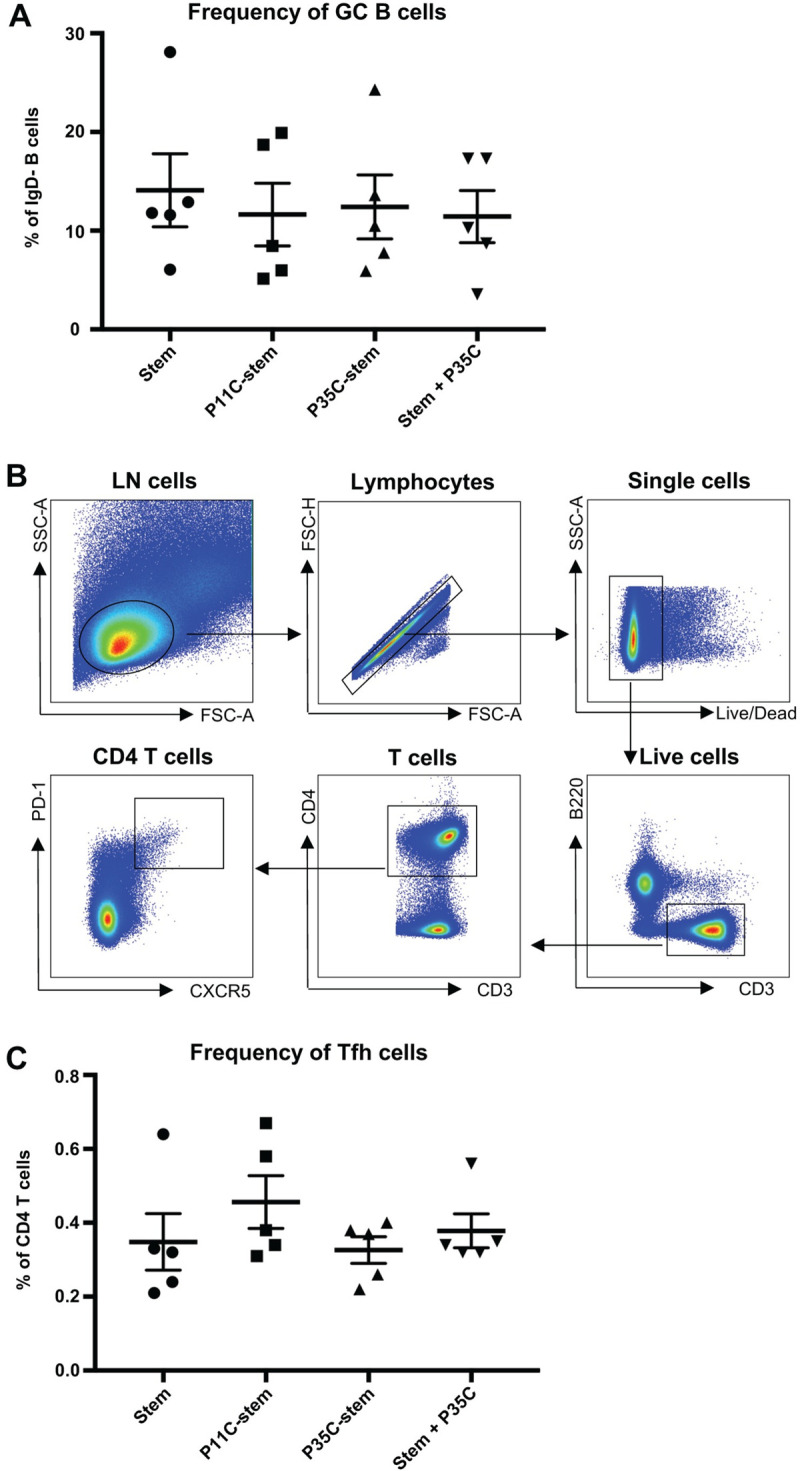
Bulk GC B and Tfh cell responses in immunized mice. (A) Frequency of bulk GC B cells (B220+IgD-CD38loGL7+) from mice (n = 5) immunized with adjuvanted (Addavax) immunogens. Data indicate the mean ± SEM. (B) Gating strategy for Tfh cells. Lymphocytes were identified by forward scatter area (FSC-A) and side-scatter area (SSC-A). Doublets were excluded by gating on single cells as determined by FSC-A versus FSC-H, and live cells were identified by viability dye exclusion. T cells were identified as CD3+B220- and CD4 T cells were identified as CD3+CD4+. Tfh cells were identified as CXCR5++PD-1++. For each step, the parental population is indicated above the plot. (C) Frequency of bulk Tfh cells (CD4+CXCR5++PD-1++) from mice (n = 5) immunized with adjuvanted (Addavax) immunogens. Data indicate the mean ± SEM.

We hypothesised that the enhanced stem B cell and antibody response could be due to enhanced total GC B cells or the induction of high levels of Tfh. We therefore analysed the frequency of bulk GC B cells and Tfh cells. We found that the frequency of bulk GC B cells and Tfh cells of P35C-stem immunized mice were similar to those of stem or P11C-stem or stem+P35C immunized mice (Fig 2), suggesting that P35C-stem conjugates did not drive enhanced GC activity. To investigate this further, we assessed whether P11 or P35 were CD4 T cell epitopes in influenza-infected c57BL/6 mice using our recently developed activation-induced marker (AIM) assay [23]. We found that neither P35 nor P11 peptides activated the expression of CD154 or CD25/OX40 markers above that of control in influenza-immune mice, showing they were not significant T-helper epitopes (S2 Fig). Taken together, these data demonstrated that P35C conjugation improves the immunogenicity of stem protein without increasing the frequency of total GC B cells or CD4 T-helper cells or inducing T cell help.

### P35C improves the immunogenicity of stem in the absence of adjuvants

**Fig 3:**
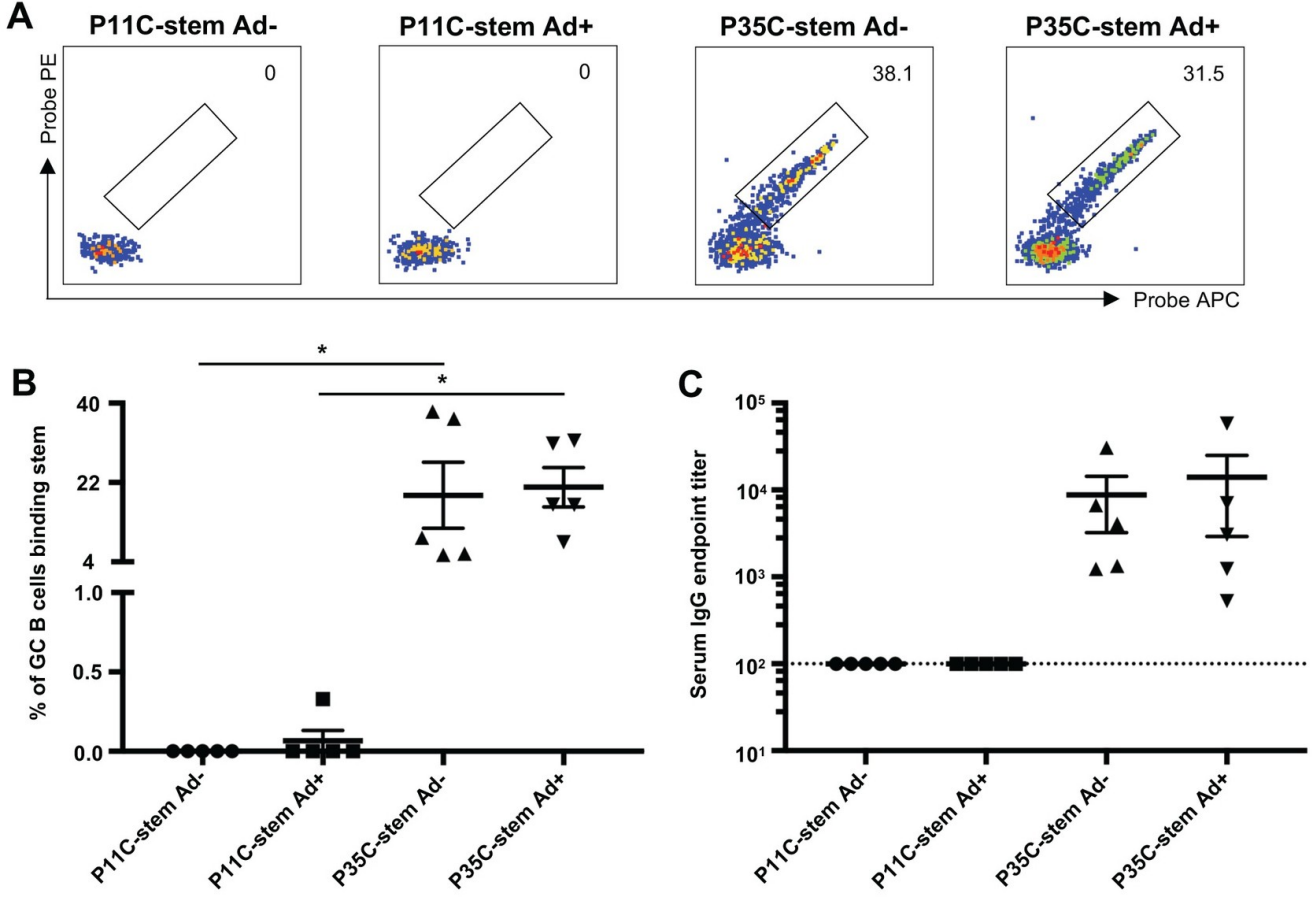
P35C improves the immunogenicity of stem in the absence of adjuvants. (A—B) Representative flow cytometric plots (A) and frequency (B) of stem-specific GC B cells from mice (n = 5) immunized with P11C-stem without Addavax (P11C-stem Ad-) or with Addavax (P11C-stem Ad+) and P35C-stem without Addavax (P35C-stem Ad-) or with Addavax (P35C-stem Ad+). (C) Serum endpoint total IgG titres of mice (n = 5) immunized with P11C-stem without Addavax (P11C-stem Ad-) or with Addavax (P11C-stem Ad+) and P35C-stem without Addavax (P35C-stem Ad-) or with Addavax (P35C-stem Ad+). Data indicate the mean ± SEM. Statistical significance was assessed by one-way ANOVA.

The above mouse P35C-stem immunization was done in the presence of a commercial adjuvant, AddaVax. The very high stem-specific GC B cells induced by the conjugated P35C suggested it may be effective in the absence of an adjuvant. To address this question, we immunized four groups of mice with P11C-stem without AddaVax (P11C-stem Ad-), P11C-stem with AddaVax (P11C-stem Ad+), P35C-stem without AddaVax (P35C-stem Ad-) and P35C-stem with AddaVax (P35C-stem Ad+) on day 0. Draining lymph nodes (dLNs) and blood were collected on day 14 post immunization for FACS and ELISA analyses. As expected, P11C-stem elicited little stem-specific B cell responses regardless of the presence of AddaVax (Fig 3). In contrast, P35C-stem induced significantly higher frequency of stem-specific GC B cells either with or without AddaVax. Stem-specific serum IgG titer of P35C-stem (either with or without AddaVax) immunized mice were higher than that of P11C-stem (either with or without AddaVax) immunized mice. Therefore, P35C-stem enhances stem-specific B cell responses in the absence of adjuvant.

### P35C improves the immunogenicity of stem by an aggregation mechanism

**Fig 4:**
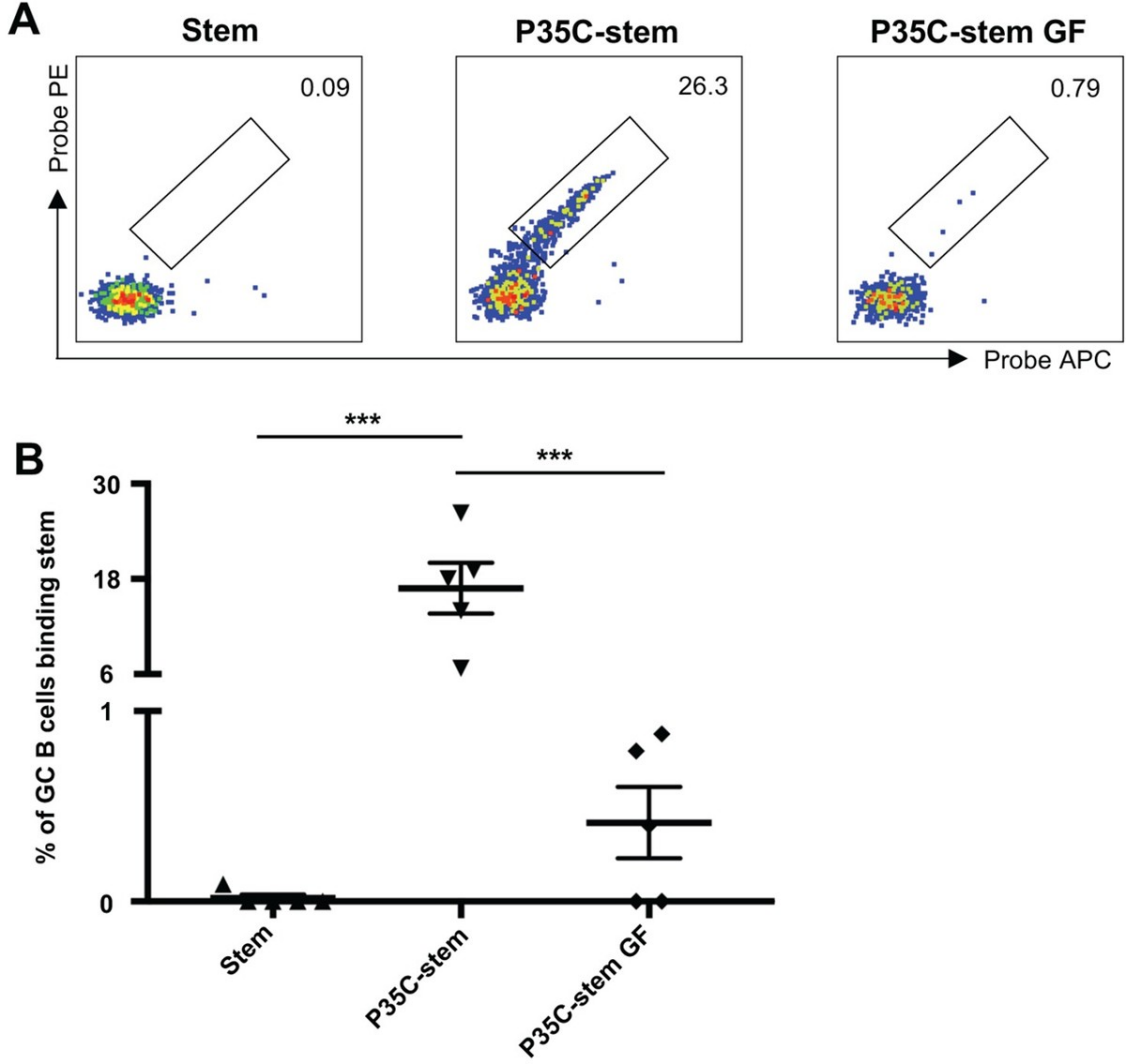
P35C improves the immunogenicity of stem by an aggregation mechanism. Representative flow cytometric plots (A) and frequency (B) of stem-specific GC B cells from mice (n = 5) immunized with adjuvanted (Addavax) PR8 HA stem, P35C-stem or gel filtrated P35C-stem (P35C-stem GF). Data indicate the mean ± SEM. Statistical significance was assessed by one-way ANOVA.

We found that P35 is not a CD4 T cell epitope (S2 Fig), and thus the enhanced stem-specific antibody responses is unlikely to be due to Tfh cell help. We therefore assessed whether the P35C-stem conjugates formed protein aggregates, which could provide T cell-independent help to B cells [15]. We filtered P35C-stem through a 0.22um filter and purified it by gel filtration after conjugation. We then immunized mice with gel-filtrated P35C-stem (P35C-stem GF) and unfiltered P35C-stem (P35C-stem). We found that the immunogenicity of gel filtrated P35C-stem significantly decreased compared with normal protocol produced P35C-stem (Fig 4). This suggests that the immunogenic components of P35C-stem were >0.22um and were removed during filtration steps.

We next performed biophysical characterisations of gel-filtrated P35C-stem and unfiltered P35C-stem through dynamic light scattering (DLS) and zeta potential experiments. The hydrodynamic size of gel-filtrated P35C-stem, as determined by the most frequent particle population in solution (hydrodynamic size by Number), was determined to be approximately 9.2 nm, similar to stem alone (10.2 nm); in contrast, the hydrodynamic size of unfiltered P35C-stem was >10,000 nm, outside the limit of distribution analysis, and so population peaks could not be resolved (S1 Table). Both samples were polydisperse, but the Z-average hydrodynamic size of the unfiltered P35C-stem (30,290 nm) was determined to be over 750-fold larger than that of the filtered conjugate (39.4 nm). Stem and PC35-stem conjugates were determined to have net negative surface charges by zeta potential analysis (S1 Table). Interestingly, the zeta potential of gel-filtrated P35C-stem was close to neutral at -2.01 mV, while the zeta potential of unfiltered P35C-stem was 10-fold larger at -20.17 mV; this indicates a larger degree of electrostatic repulsion between the unfiltered P35C-stem populations and suggests superior colloidal stability comparative to both the filtrate and stem protein itself (-3.45 mV).

This characterization suggests P35C self-aggregates after conjugation with the HA stem protein to form larger multimers which elicited superior immunity compared to soluble stem proteins.

### Generalisability of the enhanced immunogenicity of P35C conjugations to other proteins

**Fig 5:**
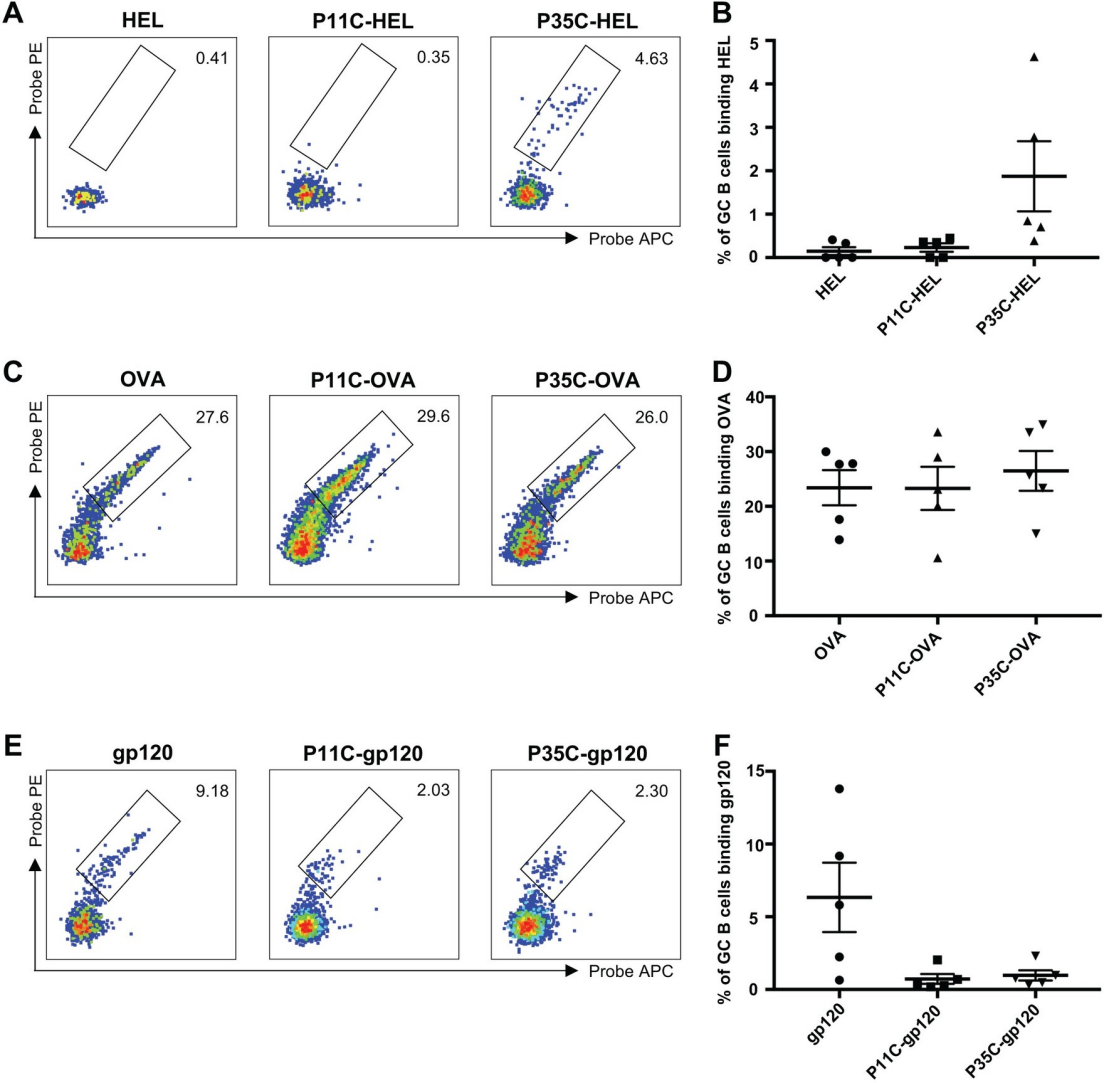
HEL/OVA/gp120-specific B cell responses in immunized mice. (A—B) Representative flow cytometric plots (A) and frequency (B) of GC B cells (B220+IgD–CD38loGL7+) from mice (n = 5) immunized with adjuvanted (Addavax) immunogens double stained with HEL probes. (C—D) Representative flow cytometric plots (C) and frequency (D) of GC B cells (B220+IgD–CD38loGL7+) from mice (n = 5) immunized with adjuvanted (Addavax) immunogens double stained with OVA probes. (E—F) Representative flow cytometric plots (E) and frequency (F) of GC B cells (B220+IgD–CD38loGL7+) from mice (n = 5) immunized with adjuvanted (Addavax) immunogens double stained with gp120 probes. Data indicate the mean ± SEM.

The unfiltered P35C-stem was highly immunogenic and we hypothesised that this phenomenon might also be true when other immunogens were conjugated to P35. To assess the generalizability of the application of P35C, we tested another immunogen, hen egg lysozyme (HEL), for conjugation with P35C and mouse immunization. Three groups of C57BL/6 mice were immunized: with HEL protein alone, with the P11C-HEL conjugate or with the P35C-HEL conjugate. Similar to results obtained with the HA stem protein alone, HEL alone elicited little or no HEL-specific B cell responses (Fig 5A and 5B). The P35C-HEL immunogen, but not the P11C-HEL immunogen, increased the frequency of HEL-specific GC B cells, although it was not statistically significant.

To further probe the ability of P35C conjugation to improve humoral immunity, we selected another well-studied and immunogenic model antigen, OVA, for conjugation with P35C and mouse immunization. C57BL/6 mice were immunized with OVA protein alone or P11C-OVA conjugate or P35C-OVA conjugate. Compared with the HA stem and HEL proteins, OVA was more immunogenic and elicited a strong OVA-specific B cell responses in the absence of peptide conjugation (Fig 5C and 5D). After conjugation with either P35C or the control P11C peptide, the new immunogens did not significantly increase frequency of OVA-specific GC B cells over their already high levels.

Next, we selected another immunogen, the HIV-1 Env monomer gp120, for conjugation with P35C and mouse immunization. C57BL/6 mice were immunized with gp120 protein alone or P11C-gp120 conjugate or P35C-gp120 conjugate. gp120 alone elicited reasonable gp120-specific B cell responses (Fig 5E and 5F). Surprisingly, gp120-specific GC B cells of P11C-gp120 or P35C-gp120 immunized mice were significantly lower than gp120 alone immunized mice. Since peptide and protein conjugation using Sulfo-SMCC is not site-specific, we surmise that P11C and P35C might block part of the original B cell epitopes of gp120 itself and damage gp120-specific B cell responses.

## Discussion

Synthetic oligopeptides have displayed significant adjuvant potential in animal studies recent years [25–28]. Here we showed that conjugation of a short peptide, P35C, improved the immunogenicity of weakly immunogenic HA stem protein in C57BL/6 mice. P35 is 17-amino acid peptide derived from the head domain of PR8 HA. Previous studies demonstrated that glutamine/asparagine (Q/N)-rich domains have a high tendency to form aggregates [29, 30]. P35C is also rich in Q/N-, including two Q and three N and we speculate that this is probably the chemical basis for P35C forming aggregates.

Tan et al. from our group recently demonstrated that HA stem was a poor immunogen which elicited little or no stem-specific antibody response in C57BL/6 mice [10]. In the present study, we found that P35C-stem conjugates significantly enhanced stem-specific antibody responses independent of Tfh cell help. Although P35 is not a strong CD4 T cell epitope in C57BL/6 mice (S2 Fig), we cannot rule out that a weak response below the threshold of detection could be present. To more definitively confirm that the enhanced immunogenicity is T cell-independent, the immunogenicity of stem and P35C-stem could be compared in TCR-deficient mice in future studies. The improved immunogenicity of stem is likely due to P35C induced aggregation of stem proteins since filtration abrogated this improved immunogenicity. Protein aggregates are known to be more efficient in eliciting immune responses than their monomeric forms even in the absence of T-cell help [15, 31]. The enhanced stem-specific antibody responses is probably related to the ability of extensive cross-linking of B-cell receptor by multiple stem proteins displayed on the surface of P35C-stem aggregates [15, 32]. Another possible mechanism would resemble the depot effect of aluminium-containing adjuvants [33–35]. Aggregated stem proteins are trapped within the site of injection and tend to be relatively stabilized against degradation, which allows prolonged release of antigens [25, 27, 32]. The slow release of antigens could provide continual stimulation to B cells directly and/or more durable peptide substrate presented by APC to T cells [10, 27]. In addition, it is possible that aggregated stem proteins promote phagocytosis of antigens by antigen-presenting cells since particulate or aggregated forms of antigens are more efficiently taken up and presented by APC than soluble proteins [18, 28, 36, 37]. Although we showed that P35C-stem improved stem-specific B cell and serological responses, whether the enhanced antibody responses induced by P35C-stem can neutralize the infectivity of live virus and confer protection of mice against influenza virus infection remain to be investigated in future studies.

To investigate whether the enhanced immunogenicity of stem protein by P35C conjugation is generalizable, we tested another immunogen, HEL, for conjugation with P35C and mouse immunization. Our data in groups of 5 mice showed that P35C-HEL conjugates could increase the frequency of HEL-specific GC B cells and HEL-specific serum IgG titre. We also conjugated P35C with HIV gp120 protein and OVA protein and elevated their immune immunogenicity in mice. We found that P35C-gp120 conjugates or P35C-OVA conjugates did not increase gp120-specific or OVA-specific antibody responses compared with soluble gp120 protein or OVA protein. Possible reasons are: 1) since peptide and protein conjugation using Sulfo-SMCC is not site-specific, P35C might block some of the original B cell epitopes of gp120 or OVA protein; 2) protein aggregation can cover some of the original B cell epitopes of gp120 or OVA protein. Either scenario could limit gp120-specific or OVA-specific B cell responses and offset the enhancement effect of P35C induced aggregation. This is consistent with previous studies that, in some cases, aggregation of a protein antigen did not increase, or even reduced, immunogenicity due to loss of immunogenic epitopes [16].

Conjugation of a highly immunogenic carrier protein with a poor immunogen is an effective strategy to rescue its immunogenicity [38]. However, because of the limited number of potent carrier proteins, conjugate vaccines against various pathogens may be coupled with the same carrier [39]. In addition, repeated immunization is commonly required to induce adequate level of protective immune responses even for the same conjugate vaccine. Pre-existing immunity to the carrier may suppress subsequent immune responses to the same vaccine upon booster immunization or to another vaccine coupled with the same carrier; this phenomenon is termed carrier induced epitopic suppression (CIES) [39, 40]. CIES may happen through competition between carrier-specific and hapten-specific B cells for the antigen or suppression by carrier-induced regulatory T cells [39–41]. Although P35C may also induce anti-P35C immune responses, the level of P35C-specific immune responses will be very low, if any. Thus, use of P35 as a “carrier” for stem protein can probably avoid the potential concern of CIES. In summary, we showed that P35C conjugation rescued the poor immunogenicity of HA stem. P35C conjugation represents a new pathway to boost stem-specific antibody responses without introducing exotic carrier proteins which will elicit anti-carrier responses. Recombinant HA protein vaccine (Flublok) produced in the baculovirus-insect cell system has been licensed for the prevention of seasonal influenza in adults aged 18–49 years [42]. The breadth of the immune responses induced by Flublok is still narrow since the mode of action of Flublok is similar to that of traditional egg-based inactivated vaccines [43]. Stem-based influenza vaccine which can elicit immune responses to a broader range of influenza viruses could be an important supplement to broaden the immunogenicity of current seasonal influenza vaccine, particularly in the case of an emerging pandemic.

## Supporting information

S1 Fig Binding activity of anti-HA stem monoclonal antibodies with PR8 HA stem or P35C-stem. Binding activity of anti-HA stem monoclonal antibodies CR9114 (A), TN1F11 (B), TN1G09 (C) and control anti-HIV-1 gp120 antibody VRC01 (D) with PR8 HA stem (red line) or P35C-stem (blue line) measured by ELISA. Titration curves were generated using sigmoid dose-response of nonlinear fit with GraphPad Prism.
(TIF)

S2 Fig Activation-induced marker expression of mLN cells from influenza-infected mice following stimulation. (A) Gating strategy to identify CD4 T cells in the mLN. Lymphocytes were identified by forward scatter area (FSC-A) and side-scatter area (SSC-A). Doublets were excluded by gating on single cells as determined by FSC-A versus FSC-H, and live cells were identified by viability dye exclusion. T cells were identified as CD3+B220- and CD4 T cells were identified as CD3+CD4+. For each step, the parental population is indicated above the plot. (B) Representative flow cytometric plots of CD154 expression on CD4 T cells after DMSO, P11, P35, or Con A stimulation for 18 h. (C) Representative flow cytometric plots of CD25/OX40 expression on CD4 T cells after DMSO, P11, P35, or Con A stimulation for 18 h.
(TIF)

S1 Table Biophysical characterisation of stem protein and P35C-stem conjugates. (DOCX)

## References

[1] Krammer F, Smith GJD, Fouchier RAM, Peiris M, Kedzierska K, Doherty PC, et al Influenza. Nat Rev Dis Primers. 2018;4(1):3 Epub 2018/06/30. 10.1038/s41572-018-0002-y . 29955068 PMC7097467

[2] Chen JR, Liu YM, Tseng YC, Ma C. Better influenza vaccines: an industry perspective. J Biomed Sci. 2020;27(1):33 Epub 2020/02/16. 10.1186/s12929-020-0626-6 32059697 PMC7023813

[3] Centers for Disease Control and Prevention. Past seasons vaccine effectiveness estimates. Adjusted vaccine effectiveness estimates for influenza seasons from 2004–2018. https://www.cdc.gov/flu/vaccines-work/past-seasons-estimates.html. Accessed 9 April 2020.

[4] Ostrowsky J, Arpey M, Moore K, Osterholm M, Friede M, Gordon J, et al Tracking progress in universal influenza vaccine development. Curr Opin Virol. 2020;40:28–36. Epub 2020/04/13. 10.1016/j.coviro.2020.02.003 . 32279026

[5] David M. Knipe PMH. Orthomyxoviridae Fields Virology, Sixth Edition 2013.

[6] Robert G. Webster ASM, Braciale Thomas J., Lamb Robert A. Structure and replication Textbook of Influenza, Second Edition 2013.

[7] Pica N, Palese P. Toward a universal influenza virus vaccine: prospects and challenges. Annu Rev Med. 2013;64:189–202. Epub 2013/01/19. 10.1146/annurev-med-120611-145115 . 23327522

[8] Nachbagauer R, Palese P. Is a Universal Influenza Virus Vaccine Possible? Annu Rev Med. 2020;71:315–27. Epub 2019/10/11. 10.1146/annurev-med-120617-041310 . 31600454

[9] Sui J, Hwang WC, Perez S, Wei G, Aird D, Chen LM, et al Structural and functional bases for broad-spectrum neutralization of avian and human influenza A viruses. Nat Struct Mol Biol. 2009;16(3):265–73. Epub 2009/02/24. 10.1038/nsmb.1566 19234466 PMC2692245

[10] Tan HX, Jegaskanda S, Juno JA, Esterbauer R, Wong J, Kelly HG, et al Subdominance and poor intrinsic immunogenicity limit humoral immunity targeting influenza HA stem. J Clin Invest. 2019;129(2):850–62. Epub 2018/12/07. 10.1172/JCI123366 30521496 PMC6355240

[11] Vogel OA, Manicassamy B. Broadly Protective Strategies Against Influenza Viruses: Universal Vaccines and Therapeutics. Front Microbiol. 2020;11:135 Epub 2020/03/03. 10.3389/fmicb.2020.00135 32117155 PMC7020694

[12] Snapper CM. Distinct Immunologic Properties of Soluble Versus Particulate Antigens. Front Immunol. 2018;9:598 Epub 2018/04/06. 10.3389/fimmu.2018.00598 29619034 PMC5871672

[13] Irvine DJ, Read BJ. Shaping humoral immunity to vaccines through antigen-displaying nanoparticles. Curr Opin Immunol. 2020;65:1–6. Epub 2020/03/23. 10.1016/j.coi.2020.01.007 . 32200132 PMC7501207

[14] Yassine HM, Boyington JC, McTamney PM, Wei CJ, Kanekiyo M, Kong WP, et al Hemagglutinin-stem nanoparticles generate heterosubtypic influenza protection. Nat Med. 2015;21(9):1065–70. Epub 2015/08/25. 10.1038/nm.3927 . 26301691

[15] Rosenberg AS. Effects of protein aggregates: an immunologic perspective. AAPS J. 2006;8(3):E501–7. Epub 2006/10/10. 10.1208/aapsj080359 17025268 PMC2761057

[16] Wang W, Singh SK, Li N, Toler MR, King KR, Nema S. Immunogenicity of protein aggregates—concerns and realities. Int J Pharm. 2012;431(1–2):1–11. Epub 2012/05/02. 10.1016/j.ijpharm.2012.04.040 . 22546296

[17] Hermeling S, Schellekens H, Maas C, Gebbink MF, Crommelin DJ, Jiskoot W. Antibody response to aggregated human interferon alpha2b in wild-type and transgenic immune tolerant mice depends on type and level of aggregation. J Pharm Sci. 2006;95(5):1084–96. Epub 2006/03/23. 10.1002/jps.20599 . 16552750

[18] Ilyinskii PO, Thoidis G, Sherman MY, Shneider A. Adjuvant potential of aggregate-forming polyglutamine domains. Vaccine. 2008;26(26):3223–6. Epub 2008/05/10. 10.1016/j.vaccine.2008.03.078 . 18467011

[19] Wei CJ, Xu L, Kong WP, Shi W, Canis K, Stevens J, et al Comparative efficacy of neutralizing antibodies elicited by recombinant hemagglutinin proteins from avian H5N1 influenza virus. J Virol. 2008;82(13):6200–8. Epub 2008/04/18. 10.1128/JVI.00187-08 18417563 PMC2447076

[20] Moyer TJ, Kato Y, Abraham W, Chang JYH, Kulp DW, Watson N, et al Engineered immunogen binding to alum adjuvant enhances humoral immunity. Nat Med. 2020;26(3):430–40. Epub 2020/02/19. 10.1038/s41591-020-0753-3 32066977 PMC7069805

[21] Morefield GL, HogenEsch H, Robinson JP, Hem SL. Distribution of adsorbed antigen in mono-valent and combination vaccines. Vaccine. 2004;22(15–16):1973–84. Epub 2004/05/04. 10.1016/j.vaccine.2003.10.040 . 15121310

[22] HogenEsch H, O'Hagan DT, Fox CB. Optimizing the utilization of aluminum adjuvants in vaccines: you might just get what you want. NPJ Vaccines. 2018;3:51 Epub 2018/10/17. 10.1038/s41541-018-0089-x 30323958 PMC6180056

[23] Jiang W, Wragg KM, Tan HX, Kelly HG, Wheatley AK, Kent SJ, et al Identification of murine antigen-specific T follicular helper cells using an activation-induced marker assay. J Immunol Methods. 2019;467:48–57. Epub 2019/02/26. 10.1016/j.jim.2019.02.008 . 30802449

[24] Kelly HG, Tan HX, Juno JA, Esterbauer R, Ju Y, Jiang W, et al Self-assembling influenza nanoparticle vaccines drive extended germinal center activity and memory B cell maturation. JCI Insight. 2020;5(10). Epub 2020/05/22. 10.1172/jci.insight.136653 32434990 PMC7259527

[25] Rudra JS, Tian YF, Jung JP, Collier JH. A self-assembling peptide acting as an immune adjuvant. Proc Natl Acad Sci U S A. 2010;107(2):622–7. Epub 2010/01/19. 10.1073/pnas.0912124107 20080728 PMC2818904

[26] Saenz R, Souza Cda S, Huang CT, Larsson M, Esener S, Messmer D. HMGB1-derived peptide acts as adjuvant inducing immune responses to peptide and protein antigen. Vaccine. 2010;28(47):7556–62. Epub 2010/08/31. 10.1016/j.vaccine.2010.08.054 20800114 PMC2963688

[27] Grenfell RF, Shollenberger LM, Samli EF, Harn DA. Vaccine self-assembling immune matrix is a new delivery platform that enhances immune responses to recombinant HBsAg in mice. Clin Vaccine Immunol. 2015;22(3):336–43. Epub 2015/01/23. 10.1128/CVI.00714-14 25609075 PMC4340892

[28] Jones JC, Settles EW, Brandt CR, Schultz-Cherry S. Virus aggregating peptide enhances the cell-mediated response to influenza virus vaccine. Vaccine. 2011;29(44):7696–703. Epub 2011/08/16. 10.1016/j.vaccine.2011.07.133 21839131 PMC3190079

[29] Wang X, Das TK, Singh SK, Kumar S. Potential aggregation prone regions in biotherapeutics: A survey of commercial monoclonal antibodies. MAbs. 2009;1(3):254–67. Epub 2010/01/13. 10.4161/mabs.1.3.8035 20065649 PMC2726584

[30] Michelitsch MD, Weissman JS. A census of glutamine/asparagine-rich regions: implications for their conserved function and the prediction of novel prions. Proc Natl Acad Sci U S A. 2000;97(22):11910–5. Epub 2000/10/26. 10.1073/pnas.97.22.11910 11050225 PMC17268

[31] Sauerborn M, Brinks V, Jiskoot W, Schellekens H. Immunological mechanism underlying the immune response to recombinant human protein therapeutics. Trends Pharmacol Sci. 2010;31(2):53–9. Epub 2009/12/08. 10.1016/j.tips.2009.11.001 . 19963283

[32] Moyer TJ, Zmolek AC, Irvine DJ. Beyond antigens and adjuvants: formulating future vaccines. J Clin Invest. 2016;126(3):799–808. Epub 2016/03/02. 10.1172/JCI81083 26928033 PMC4767337

[33] Ghimire TR. The mechanisms of action of vaccines containing aluminum adjuvants: an in vitro vs in vivo paradigm. Springerplus. 2015;4:181 Epub 2015/05/02. 10.1186/s40064-015-0972-0 25932368 PMC4406982

[34] Martinon S, Cisneros A, Villicana S, Hernandez-Miramontes R, Mixcoha E, Calderon-Vargas P. Chemical and Immunological Characteristics of Aluminum-Based, Oil-Water Emulsion, and Bacterial-Origin Adjuvants. J Immunol Res. 2019;2019:3974127 Epub 2019/06/18. 10.1155/2019/3974127 31205956 PMC6530223

[35] He P, Zou Y, Hu Z. Advances in aluminum hydroxide-based adjuvant research and its mechanism. Hum Vaccin Immunother. 2015;11(2):477–88. Epub 2015/02/19. 10.1080/21645515.2014.1004026 25692535 PMC4514166

[36] Bachmann MF, Jennings GT. Vaccine delivery: a matter of size, geometry, kinetics and molecular patterns. Nat Rev Immunol. 2010;10(11):787–96. Epub 2010/10/16. 10.1038/nri2868 . 20948547

[37] Kovacsovics-Bankowski M, Clark K, Benacerraf B, Rock KL. Efficient major histocompatibility complex class I presentation of exogenous antigen upon phagocytosis by macrophages. Proc Natl Acad Sci U S A. 1993;90(11):4942–6. Epub 1993/06/01. 10.1073/pnas.90.11.4942 8506338 PMC46629

[38] Rappuoli R, De Gregorio E, Costantino P. On the mechanisms of conjugate vaccines. Proc Natl Acad Sci U S A. 2019;116(1):14–6. Epub 2018/12/24. 10.1073/pnas.1819612116 30578318 PMC6320500

[39] Jegerlehner A, Wiesel M, Dietmeier K, Zabel F, Gatto D, Saudan P, et al Carrier induced epitopic suppression of antibody responses induced by virus-like particles is a dynamic phenomenon caused by carrier-specific antibodies. Vaccine. 2010;28(33):5503–12. Epub 2010/03/24. 10.1016/j.vaccine.2010.02.103 . 20307591

[40] McCluskie MJ, Evans DM, Zhang N, Benoit M, McElhiney SP, Unnithan M, et al The effect of preexisting anti-carrier immunity on subsequent responses to CRM197 or Qb-VLP conjugate vaccines. Immunopharmacol Immunotoxicol. 2016;38(3):184–96. Epub 2016/04/29. 10.3109/08923973.2016.1165246 . 27121368

[41] Dagan R, Poolman J, Siegrist CA. Glycoconjugate vaccines and immune interference: A review. Vaccine. 2010;28(34):5513–23. Epub 2010/07/06. 10.1016/j.vaccine.2010.06.026 . 20600514

[42] Yang LP. Recombinant trivalent influenza vaccine (flublok((R))): a review of its use in the prevention of seasonal influenza in adults. Drugs. 2013;73(12):1357–66. Epub 2013/08/10. 10.1007/s40265-013-0103-6 . 23928902

[43] Cox MM, Izikson R, Post P, Dunkle L. Safety, efficacy, and immunogenicity of Flublok in the prevention of seasonal influenza in adults. Ther Adv Vaccines. 2015;3(4):97–108. Epub 2015/10/20. 10.1177/2051013615595595 26478817 PMC4591523

