# Supplementary figures and images for: Aggregation by peptide conjugation rescues poor immunogenicity of the HA stem
Source: PLoS One. 2020 Nov 2;15(11):e0241649. doi: 10.1371/journal.pone.0241649 (PMC7605677; doi:10.1371/journal.pone.0241649)

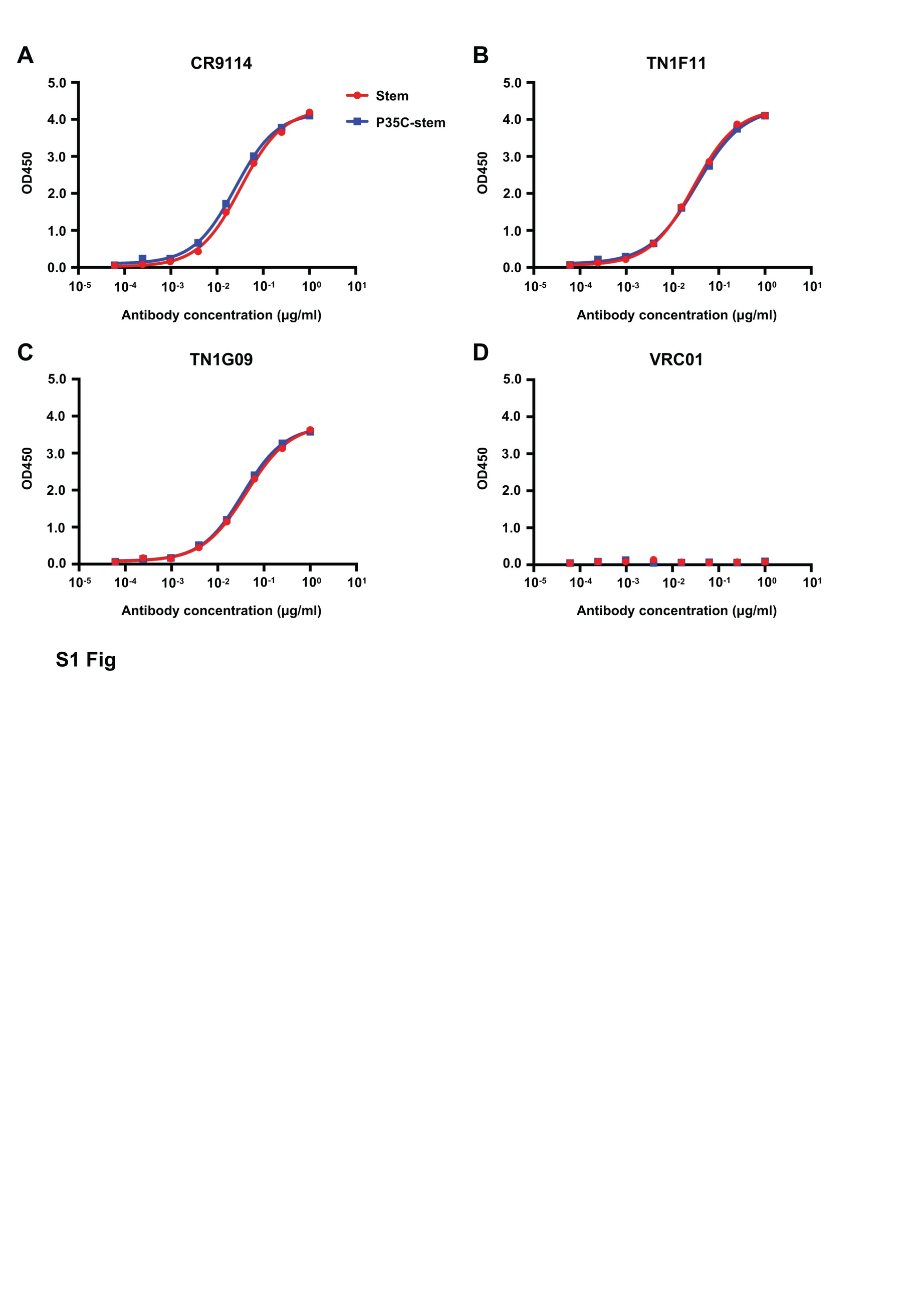

Supplement: S1 Fig — Binding activity of anti-HA stem monoclonal antibodies CR9114 (A), TN1F11 (B), TN1G09 (C) and control anti-HIV-1 gp120 antibody VRC01 (D) with PR8 HA stem (red line) or P35C-stem (blue line) measured by ELISA. Titration curves were generated using sigmoid dose-response of nonlinear fit with GraphPad Prism. (TIF) [file pone.0241649.s001.tif]

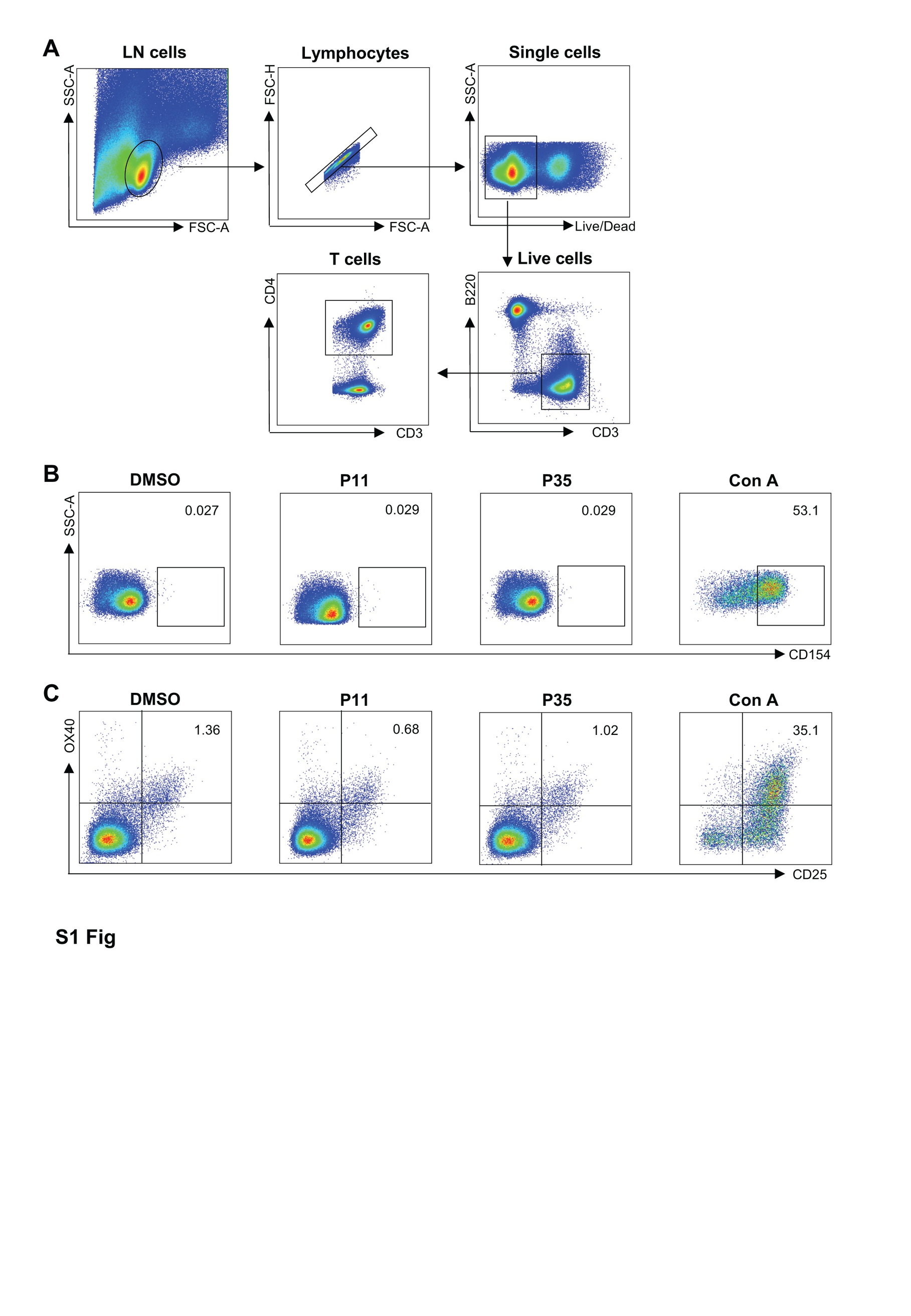

Supplement: S2 Fig — (A) Gating strategy to identify CD4 T cells in the mLN. Lymphocytes were identified by forward scatter area (FSC-A) and side-scatter area (SSC-A). Doublets were excluded by gating on single cells as determined by FSC-A versus FSC-H, and live cells were identified by viability dye exclusion. T cells were identified as CD3+B220- and CD4 T cells were identified as CD3+CD4+. For each step, the parental population is indicated above the plot. (B) Representative flow cytometric plots of CD154 expression on CD4 T cells after DMSO, P11, P35, or Con A stimulation for 18 h. (C) Representative flow cytometric plots of CD25/OX40 expression on CD4 T cells after DMSO, P11, P35, or Con A stimulation for 18 h. (TIF) [file pone.0241649.s002.tif]
