# Supplementary material for: Aggregation by peptide conjugation rescues poor immunogenicity of the HA stem
Source: PLoS One. 2020 Nov 2;15(11):e0241649. doi: 10.1371/journal.pone.0241649 (PMC7605677; doi:10.1371/journal.pone.0241649)
Supplement: S1 Table — (DOCX) [file pone.0241649.s003.docx]

**S1 Table. Biophysical characterisation of stem protein and P35C-stem conjugates.**

| **Sample** | **Polydispersity index** | **Hydrodynamic size by Number (nm)** | **Z-average hydrodynamic size (nm)** | **Zeta potential (mV)** |
| --- | --- | --- | --- | --- |
| **Stem** | 0.266 ± 0.111 | 10.2 ± 0.2 | 145.5 ± 74.6 | -3.45 ± 0.345 |
| **P35C-stem (filtered)** | 0.588 ± 0.097 | 9.2 ± 0.6 | 39.4 ± 9.6 | -2.01 ± 0.919 |
| **P35C-stem (unfiltered)** | 0.733 ± 0.064 | >10,000* | 30290 ± 2257.8 | -20.2 ± 1.79 |

*unable to resolve due to size exceeding limit (10,000 nm) of distribution analysis model
